# Normative hippocampal volumetric measurements using magnetic resonance imaging

**DOI:** 10.3906/sag-1903-233

**Published:** 2019-10-24

**Authors:** Meltem ÖZDEMİR, Handan SOYSAL, Önder ERASLAN, Alper DİLLİ

**Affiliations:** 1 Department of Radiology, Dışkapı Yıldırım Beyazıt Health Application and Research Center, Medical Sciences University, Ankara, Turkey; 2 Department of Anatomy, Faculty of Dentistry, Yıldırım Beyazıt University, Ankara, Turkey

**Keywords:** Hippocampal volume, normative data, epilepsy, dementia

## Abstract

**Background/aim:**

A wide variety of neurological and psychiatric disorders have been shown to be closely related to changes in hippocampal volume (HV). It appears that hippocampal volumetry will be an indispensable part of clinical practice for a number of neuropsychiatric disorders in the near future. The aim of this study was to establish a normative data set for HV according to age and sex in the general population.

**Materials and methods:**

Hippocampal magnetic resonance imaging scans of 302 healthy volunteers were obtained using a 1.5 T unit with a 20-channel head coil. The hippocampal volumetric assessment was conducted using the volBrain fully automated segmentation algorithm on coronal oblique T1-weighted magnetization prepared rapid gradient-echo (MP-RAGE) images obtained perpendicular to the long axis of the hippocampus. The mean values of HV of groups according to age and sex were calculated. The associations between HV and age and sex were analyzed.

**Results:**

The mean HV of the study group was found to be 3.81 ± 0.46 cm3. We found that the mean HV of males (3.94 ± 0.49 cm3) was significantly higher than that of females (3.74 ± 0.42 cm3), and the mean right HV (3.86 ± 0.48 cm3) was significantly higher than that of the left HV (3.78 ± 0.49 cm3) (P = 0.001). Among both females and males, there were statistically significant but poor negative correlations between age and volumetric measurements of both the right and the left hippocampi (P < 0.05).

**Conclusion:**

The normative hippocampal volumetric data obtained in this study may be beneficial in clinical applications for many neuropsychiatric diseases, especially for mesial temporal sclerosis and cognitive disorders.

## 1. Introduction

The hippocampus, a small anatomical region with a unique shape, is located medially in the temporal lobe and situated underneath the cortex. It is made up of complex bilaminar gray matter which plays a crucial role in cognition, especially in memory processes [1]. Its involvement in episodic, semantic, and spatial memory processes, and its relevance in a number of neurological and psychiatric disorders, has led to the hippocampus remaining at the focus of neuroimaging research for many years [2]. Furthermore, a recent functional magnetic resonance imaging (MRI) study conducted by Chan et al. revealed that low-frequency activities of the hippocampus can maintain brainwide functional connectivity in the cerebral cortex, and enhance the responsiveness of the brain [3]. These findings imply that this fascinating anatomical region could be the functional center of the whole brain.

MRI is the method of choice in qualitative and quantitative evaluation of the hippocampus. The main clinical indications requiring volumetric assessment of the region are intractable epilepsy and cognitive disorders [1]. Hippocampal volumetry has become an important element of diagnostic as well as prognostic evaluations of these diseases [4–6]. Moreover, the results of recent neuroimaging studies suggest that hippocampal volumetry may be an indispensable part of clinical applications in many other neuropsychiatric disorders in the near future [7–13].

To date, many studies have reported normative data for the hippocampal volume (HV) in the relevant literature [14–22]. However, the previously reported hippocampal volumetric data show variations among studies. In addition, the reported volumetric findings show inconsistency and controversion for many disorders. Large variations between chosen anatomical protocols for the manual delineation of the hippocampus in MRI sections have been addressed as one of the main sources of these incompatibilities. Recently, various semi- and fully automated techniques have been introduced to segment regional brain structures in order to overcome the limitations of the manual segmentation method [23]. The aim of this study was to establish a normative data set for HV according to age and sex in adult population, using a newly-introduced fully automated segmentation algorithm.

## 2. Materials and methods

### 2.1. Patient population

The approval of the institutional review board was received before the execution of this work began. A signed form indicating that the patient was informed and had consented was received from each participant. The study was conducted between 1 June 2018 and 1 February 2019. The study group consisted of healthy volunteers with no history of surgical treatment or trauma of the brain, neurological or psychiatric disease, or substance abuse. A mini-score assessment was performed in order to rule out psychiatric disease as well as cognitive impairment. Our study group consisted of 302 participants. There were 118 men and 184 women with a mean age of 45.16 ± 17.62 years (range: 11–84). 

### 2.2. MRI protocol and segmentation method

**Figure 1:**
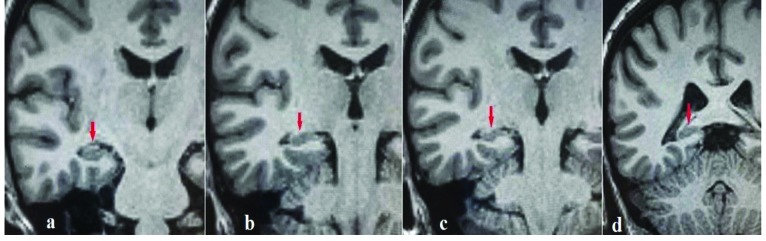
Consecutive coronal oblique T1-weighted MP-RAGE images demonstrating thehippocampal head (a), body (b, c), and tail (d) (red arrows).

MRI of the hippocampus was performed using a 1.5 T unit (Magnetom Aera, Siemens, Erlangen, Germany) with a 20-channel head coil. The hippocampal volumetric assessment was conducted on coronal oblique T1-weighted images obtained perpendicular to the long axis of the hippocampus. MP-RAGE sequence was used with the following parameters: TR = 2400 ms, TE = 3.54 ms, FOV = 240 mm, slice thickness = 1.2 mm, voxel size = 1.3 × 1.3 × 1.2 mm (Figure 1). 

**Figure 2:**
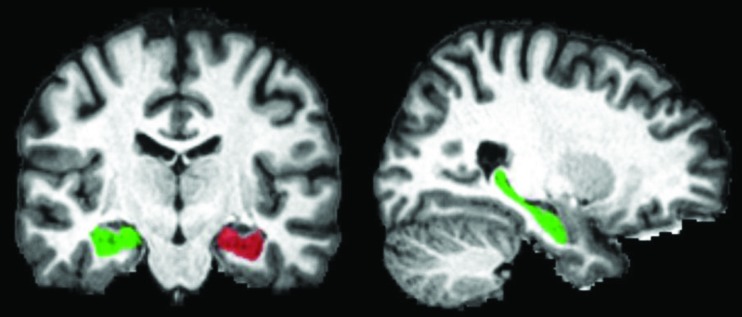
Fully automated hippocampal segmentation by volBrain.

**Figure 3:**
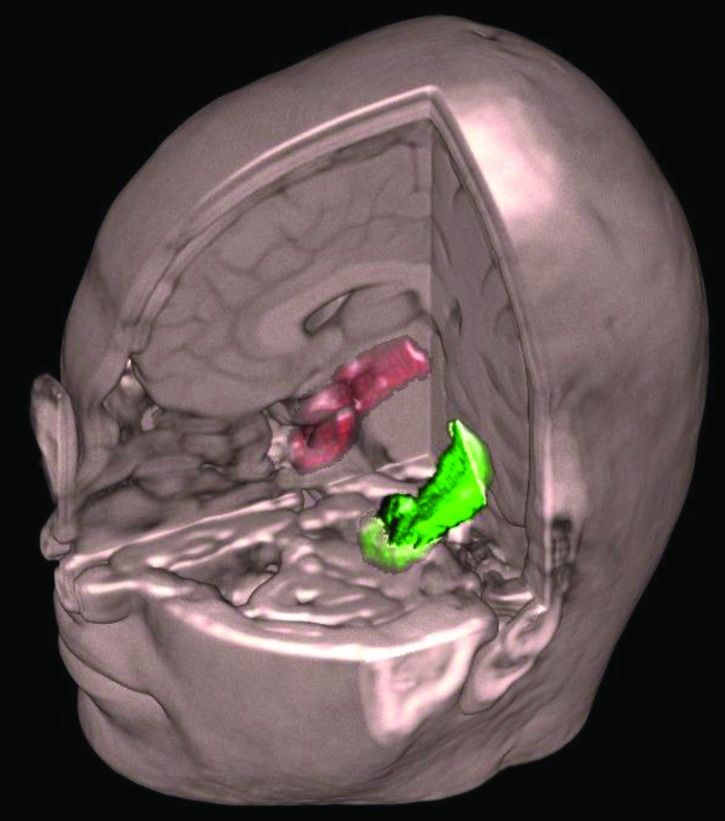
3D visualization of volBrain data.

MRI data processing and hippocampal volumetric analyses were performed using volBrain (v.1.0, http://volbrain.upv.es), a free online MRI brain volumetry system. VolBrain is a fully automated segmentation technique of which the algorithm is based on multiatlas patch-based label fusion segmentation technology (Figures 2 and 3) [24]. 

### 2.3. Statistical analysis

The normality of distribution of continuous variables was tested with the Shapiro–Wilk test. The Wilcoxon test was used to compare the left and right HV measurements of the same subjects, and the Mann–Whitney U test was used to compare the sexes for nonnormal data. Furthermore, the Kruskal–Wallis test and Dunn multiple comparison tests were performed to compare nonnormal numerical variables across age groups. Spearman rank correlation coefficient was calculated to investigate the relationship between two numerical variables. Frequency and percentage for categorical variables and mean ± standard deviation for numerical variables are given as descriptive statistics. Statistical analysis was performed with SPSS for Windows version 24.0, and a P value <0.05 was accepted as statistically significant.

## 3. Results 

**Figure 4:**
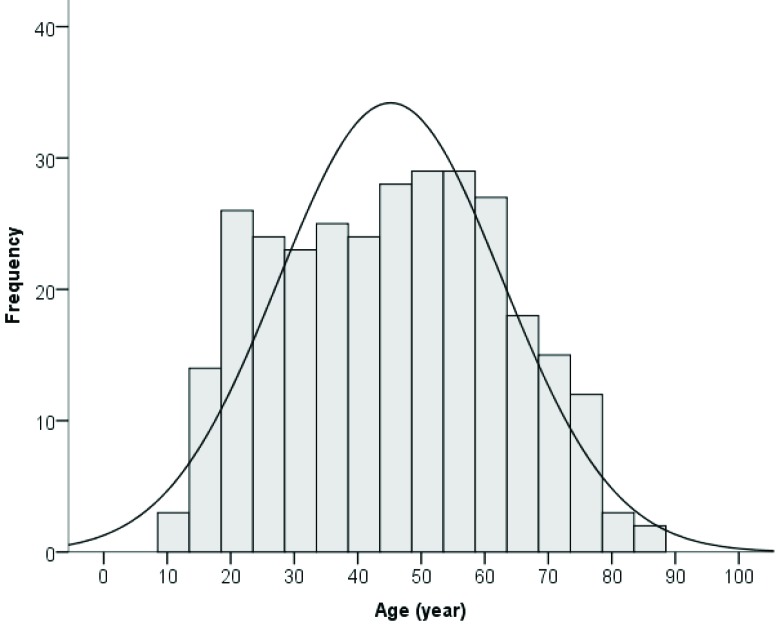
Age-frequency distribution diagram.

The age of the study population ranged between 11 and 84. Age-frequency distribution is shown in Figure 4. In the current sample of 302 participants, the mean HV was found to be 3.81 ± 0.46 cm3, with the upper and the lower limits being 5.20 cm3 and 1.92 cm3, respectively. The mean right HV was 3.86 ± 0.48 cm3 and the mean left HV was 3.78 ± 0.49 cm3, with the difference being statistically significant (P = 0.001). The mean HV in males and females were 3.94 ± 0.49 cm3 and 3.74 ± 0.42 cm3, respectively, with the difference being statistically significant (P = 0.001).

A mean right HV of 4 ± 0.5 cm3 in males and 3.76 ± 0.45 cm3 in females was recorded, with the difference being statistically significant (P = 0.001). Similarly, the mean left HV was 3.89 ± 0.57 cm3 in males and 3.71 ± 0.42 cm3 in females, with the difference being statistically significant (P = 0.001). 

**Figure 5:**
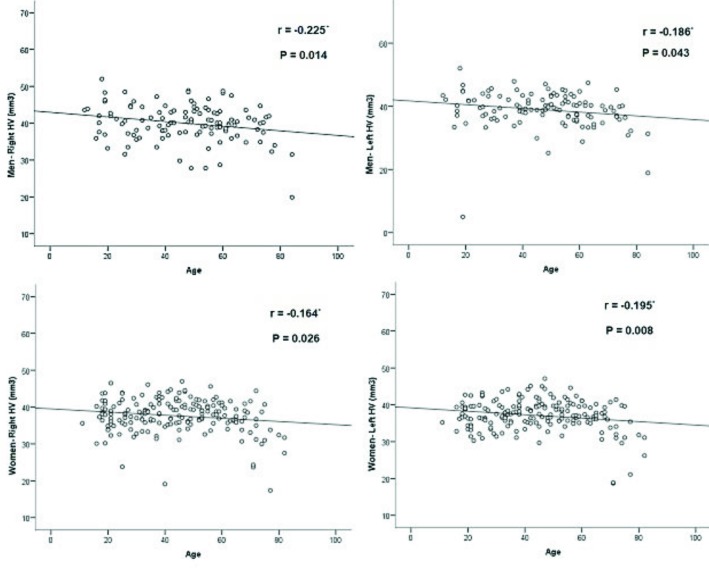
Relationship between age and volumetric measurements of the right and left hippocampi of men and women. Hippocampal volume measures show statistically significant but weak negative correlations with age in both sides of both sexes.Spearman correlation coefficient, significant at *0.05 level.

Figure 5 demonstrates the scatter plots of the HV measurements according to age for the right and left sides of males and females. For both hippocampi of both sexes, there were statistically significant but weak negative correlations between volumetric measurements and age. The mean HV measurements according to age group are demonstrated in Table. We recorded a significant difference between the mean HV of females <30 and of those >49 years of age (P = 0.001). Furthermore, we noted that the mean HV of females over the age of 69 is significantly smaller than that of younger females (P = 0.001). However, no statistically significant differences were noted between different age groups of males.

**Table:** The mean hippocampal volume measurements according to age groups.

Age interval	N	Female (mm3)		Male (mm3)		R	L	R	L
		10–19	27	3.85 ± 0.4	3.8 ± 0.23	4.3 ± 0.51	3.89 ± 1.23
20–29	44	3.7 ± 0.44	3.64 ± 0.37	4.02 ± 0.51	4.01 ± 0.39
30–39	49	3.84 ± 0.35	3.79 ± 0.33	3.94 ± 0.37	3.94 ± 0.39
40–49	55	3.84 ± 0.51	3.84 ± 0.39	4.04 ± 0.52	3.94 ± 0.49
50–59	60	3.91 ± 0.27	3.82 ± 0.28	3.98 ± 0.45	3.98 ± 0.34
60–69	37	3.74 ± 0.27	3.71 ± 0.26	4.08 ± 0.42	3.78 ± 0.48
70–84	30	3.25 ± 0.69	3.15 ± 0.71	3.67 ± 0.64	3.56 ± 0.64
P		0.002*	0.002*	0.303	0.140

R, right hippocampus; L, left hippocampus.The Kruskal–Wallis test, significant at *0.05 level.

## 4. Discussion

A wide variety of neurological and psychiatric disorders including epilepsy, cognitive impairment, depression, schizophrenia, posttraumatic stress disorder, borderline personality disorder, alcohol abuse, and Parkinson’s disease have been shown to be closely related to changes in HV [4–13]. HV loss is a well-established feature of mesial temporal sclerosis (MTS) and Alzheimer’s disease. MTS, the most common cause of temporal lobe epilepsy, is characterized by hippocampal sclerosis and atrophy. The quantification of the atrophy helps in lateralization and prognostication of seizures in patients with MTS [4]. Hippocampal volumetry helps to differentiate Alzheimer’s disease from mild cognitive impairment, and dementia from pseudodementia. It has been reported that hippocampal volumetry can also differentiate various types of dementias when used in combination with clinical and other supportive laboratory data [5,6]. 

Recent studies have revealed that bilateral HV reduction occurs in patients with depression, being more pronounced in those with recurrent disease [7]. Furthermore, it has been shown that a significant HV loss occurs in the first episode of depression, implying that HV could be a diagnostic neurobiomarker for the disease [8]. Likewise, a volume deficit in the anterior hippocampus of patients with early psychosis has been recorded in a very recent study [9]. Volume loss of the hippocampus as a whole or of its substructures is a common finding among many other recent neuroimaging studies done in neuropsychiatric populations [10–13]. These findings underline the growing need for normative hippocampal volumetric data for the benefit of clinical practice.

For the assessment of HV, the manual method as well as semiautomated and fully automated methods can be used. The manual segmentation method is currently considered the gold standard in delineating the hippocampus. Thus far, several different anatomical protocols have been adopted for manual segmentation of the region. However, it has been shown that large variations between these protocols cause inconsistency and controversion in volumetric findings in many disorders [2]. Furthermore, manual segmentation is a time-consuming method with a risk of rater bias. During the past few years, various tools for automated segmentation of brain structures have been proposed, and the use of these novel algorithms in both clinical and research settings has gained great momentum. Today, diverse automated techniques are freely or commercially available for quick and reliable segmentation of regional brain structures.

Semiautomated methods are based on the prior introduction of the knowledge of a human operator who identifies the anatomical landmarks and the boundaries [2]. Unlike the fully automated methods, the semiautomated algorithms allow the operator to edit the quality of the automatically determined segmentation. On the other hand, fully automated methods are based on either statistical shape-models or nonlinear registration to an atlas/multiple atlases. The most commonly used fully automated algorithm, FIRST (http://fsl.fmrib.ox.ac.uk), is provided as part of the FSL software library. FIRST employs models which have been generated from manually segmented standard templates, and uses a Bayesian framework which allows the probabilistic relationship of shape and intensity to be exploited in estimating volume. VolBrain (http://volbrain.upv.es) is a newly introduced fully automated segmentation technique which is based on multiatlas patch-based label fusion segmentation technology. It is a free online MRI brain volumetry system providing volumetric brain data at different scales in a web-based interface without any installation or advanced computational requirements. It is very likely that many efficient and reliable fully automated segmentation methods that work with low-cost cloud-based solutions will play an important role in volumetric brain analyses in the near future [23,24].

According to both MRI- and histology-based studies, the HV shows variations within a wide range (1.73–5.68 cm3) [15]. We found a mean HV of 3.81 cm3, with the range being 1.92–5.20 cm3 in our study population. Mohandas et al. stated that HV varies depending on ethnicity, and the HV in Western populations is higher than that of the Indian population [22]. Indeed, the reported mean HV values range between 2.78 and 4.18 cm3 according to the studies performed with European populations [15–17], whereas the range is 1.98–2.91 cm3 in those performed with Asian populations [19–21]. Mohandas et al. recorded a mean HV of 2.41 cm3 in an Indian population [22]. The volumetric results of the current study, which was conducted in a Turkish population, seem to be similar to those of the studies carried out in European populations. Consistent with previous data [14–17,21,22], we found that the HV of males is higher than that of females; for both sexes, volume of the right hippocampus is higher than that of the left one. Regardless of sex and side, the normal hippocampal volume ranges we have determined based on the findings of this study will be useful in clinical use.

In some recent volumetric studies, no statistically significant association was found between HV and age [21,22]. However, it has been shown by other studies that, although less pronounced compared to the other neuroanatomic structures, reduction of HV occurs with aging [14,20,25]. We recorded statistically significant negative correlations between age and volumetric measurements of both the right and the left hippocampi among both females and males. However, this was a weak relationship in which the mean HV difference did not reach statistical significance among the majority of the age groups.

The main limitation of this study is that the measurements were performed on a sample that included a relatively small number of participants. Further studies with larger numbers of participants are needed to validate the findings of this study. 

In conclusion, the normative data set for HV according to age and sex in an adult population obtained in this study can be beneficial in clinical applications of many neuropsychiatric diseases, foremost MTS and cognitive disorders.
